# Currency and Competence of Occupational Therapists and Consumers with Rapidly Changing Technology

**DOI:** 10.1155/2017/5612843

**Published:** 2017-01-12

**Authors:** Emily J. Steel, Ricky Buchanan, Natasha Layton, Erin Wilson

**Affiliations:** ^1^TC Beirne School of Law, The University of Queensland, St Lucia, QLD, Australia; ^2^Independent Internet Professional and Disability Activist, Melbourne, VIC, Australia; ^3^Summer Foundation/La Trobe University, Box Hill, VIC, Australia; ^4^School of Health & Social Development, Deakin University, Burwood, VIC, Australia

## Abstract

Assistive technology was once a specialised field of practice, involving products designed for populations with specific impairments or functional goals. In Australia, occupational therapists have, at times, functioned as gatekeepers to public funding, prescribing products from a predefined list. An expanding range of accessible mainstream products available via international and online markets has changed the meaning and application of assistive technology for many people with disability. In the policy context of consumer choice and cost-effectiveness, have occupational therapists been left behind? This paper describes the change in context for access to assistive technology resulting in expanded possibilities for participation and inclusion. A case study of environmental control systems is used to explore the overlap of mainstream and assistive products and the funding and services to support their uptake. The analysis describes a future policy and practice context in which assistive technology includes a spectrum of products decoupled from access to independent advice and support services. A broader scope of occupational therapy practice has potential to enhance the occupational rights of people with disability and the efficiency and effectiveness of assistive technology provision.

## 1. Introduction

Occupational therapy is founded on an understanding that people's engagement in purposeful and meaningful activities is dependent on the fit between the person, task, and environment. For people with persistent impairment or chronic conditions this translates into practices of modifying or augmenting tasks and environments to fit with an individual's capacities, thereby enhancing occupational performance [1]. Occupational therapists have traditionally applied this approach in the practice of assistive technology, involving the provision of products and services that enable individuals to participate in daily activities and life roles.

Environmental control systems allow the activation and control of electrical products remotely and are therefore valuable components of assistive solutions for people with disability [2]. Research has highlighted benefits for people who use environmental control systems including feelings of wellbeing and competence, maintenance of independence, and enablement in basic communication and entertainment [3, 4]. A grounded theory study not only found that the positive impacts of environmental control systems could be profound and “transcend” utility, but also noted that pragmatic issues (such as insufficient ports for all appliances) led to a situation where users, having become aware of what was possible, felt they were denied the full potential of this type of technology and that “transcendence of utility” was denied [5].

Mainstream products that can function as environmental control systems fit contemporary definitions of assistive technology products as “any product (including devices, equipment, instruments and software), especially produced or generally available, used by or for persons with disability: for participation; to protect, support, train, measure or substitute for body functions/structures and activities, or; to prevent impairments, activity limitations or participation restrictions” [6]. They are marketed with images of attractive couples or families in homes, promising “ambience,” “convenience,” “wellbeing,” and “possibilities.” This contrasts starkly with the stereotypes of beige equipment and clinical environments promoting “safety” and “independence” that have stigmatised assistive technology users in the past [7]. With concurrent policy reforms in Australia that focus on consumer choice and cost-effectiveness, what are the implications for future assistive technology provision systems?

This paper describes the benefits of mainstream products and ways in which they can be integrated into assistive technology policies and occupational therapy practice to meet the competing policy demands of cost-effectiveness and consumer choice, while advancing occupational rights.

## 2. Materials and Methods

This paper focuses on the experiences of one of the authors (RB) as an assistive technology user, in dialogue with the other authors as occupational therapists and assistive technology researchers. The analysis draws on and is limited to our experiences as AT users and practitioners in the context of Australian assistive technology provision systems in multiple jurisdictions. The methodology privileges the lives and rights of people with disability by regarding them as primary stakeholders and sources of knowledge [8]. It uses qualitative and interpretive methods, which have been identified as preferred ways of giving voice to the experiences of people with disability (author RB) [9]. The approach taken in this paper is consistent with the emancipatory paradigm of disability research [10] and the wider methodological principle of making the personal political that is central to the disability rights and feminism movements [11].

The case of one assistive technology user's (author RB) experiences of procuring, setting up, and using environmental control systems is used to explore changes in the product market and the implications for assistive technology practices and policy. Case-based approaches can examine multiple variables that are involved in problems related to social systems that cannot be experimentally controlled [12]. Cases are usually purposely selected to provide rich descriptions that illuminate the constructs and conditions being investigated, in this case self-direction in assistive technology provision, and would not be elicited by studying an average case or statistics [13–15]. The purpose of this case study is not to generalize an individual's experiences to other people or situations, but to examine the micro and macro contexts in which the individual's experiences are situated in order to understand the factors directly relevant to policy and practice. Data were collected from formal and informal correspondence between the authors, including via face-to-face discussion, email, and field notes. Product-related data were obtained from public websites. Ethical approval was not required for this study.

## 3. Results and Discussion

As an active and informed assistive technology user, one of the authors (RB) reflects on her own experience of selecting, customising, and using environmental control systems:

Until a few years ago anyone wanting to control a light bulb or power point remotely (e.g. from a computer or wheelchair) had very few options. Products such as the PROG were designed specifically for people with disability, and available through suppliers of “medical and disability aids”. Because these products cost in excess of $1000, they tended to be accessed by people eligible for subsidies under public health or insurance systems, necessitating the involvement of a “prescriber” responsible for assessing needs and authorising funding. As prescribers, occupational therapists were familiar with the environmental control systems available, delimited by funding and regulatory requirements (i.e. meeting relevant standards and on the approved “list”) and could assist in their set-up. Alternatively, individuals with a high level of technical know-how could purchase and set up a system such as X10 controls, but this also involved significant expense. Neither type of product was readily compatible with other assistive products commonly used, such as computers or power wheelchairs. Inexpensive, reliable and inter-operable products and systems are now available in mainstream consumer stores. These include remote-controlled light bulbs (e.g. Philips Hue) and power sockets (e.g. Belkin WeMo) that can be programmed from recipes (rather than requiring the knowledge of coding languages) and operated from smart phones, tablets or personal computers. Further advances in mainstream products that function as environmental control systems are imminent, including remote control door locks, controls for windows, curtains and blinds. Access methods will also continue to be simplified and adaptable to user preferences, such as use of voice recognition to interact with objects in the “internet of things”. These products will be no more difficult to customise and use than the smartphone software and hardware that are now ubiquitous.

Policy-makers and people with disability may question the extent to which future assistive technology provision will require professional support (or gate-keeping) [16] and public funding for assistive technology services and products or whether these can be supplied by the market [17, 18]. Our experiences (as users, occupational therapists, and researchers) suggest that assistive technology provision policies and practices in Australia are not abreast of contemporary definitions of, and markets for, assistive products. The future role of professionals such as occupational therapists may depend upon our abilities to coproduce outcomes despite pragmatic constraints [19] and our strategies to achieve assistive technology “currency” to support our clinical reasoning processes [20]. From her perspective as an occupational therapy service user and peer supporter, RB notes:

Assistive technology abandonment rates indicate that even “ideal” choosing with professional guidance is no panacea. I've heard people saying that customers should not purchase assistive products without professionals because they might make bad choices and yes, that's a risk, but the risks of being forced to use an OT are that it's expensive, there are often long wait times, your choice is usually limited by what's “on the list”, and the end result might be no more compatible with you anyway.

For the informed, experienced, and confident consumer, these changes in the mass market may negate the need for funding subsidies or occupational therapy assessments currently available through public systems. Consumers can choose products much as they would a smartphone: a consumer does not have to know about every product available on the market (of which there may be hundreds); their preferences are likely to be informed by their own experiences with product features, familiarity with brands and their compatibility with other possessions, seeing and hearing what peers are using, and testing products in shops. The decision to purchase still represents a “leap of faith” because most of these products are what economists describe as “experience goods,” meaning that their value cannot be known until they are being used [21].

Bypassing traditional assistive technology service systems enables greater consumer choice and avoids lengthy delays that are well documented [22]. However, the complexity of the mass market and its ever-changing and expanding range of products necessitate willingness to change and investment in continuous updating of knowledge and skills [23]. This is particularly important with consumer-grade environmental control systems that only last a few years and then cannot be replaced because they have been superseded, such as the i-Red infrared remote control for the iPhone. Is there support available for consumers new to using environmental controls systems or not confident selecting between products or setting them up? Some companies enhance their sales and reputation through in-store training or remote support [24, 25]. Other companies can market products at lower prices by not providing individual in-home support and follow-up, and instructions or consumer forums have not always proved to be adequate supplements for assistive technology users [26].

The risks when purchasing environmental control systems (or indeed smart phones) are comparatively less when compared to the purchase of a power wheelchair costing upwards of $4000. Thus, the cost of a “poor choice” of environmental control system is unlikely to cause significant or irreversible harm. Nonetheless, the risk of operating without expert advice or targeted information is that consumers are at the mercy of marketing. Best practice in provision of assistive products recognizes that the actual device often requires a range of “soft technologies” or human supports such as assessment, trial, adaptation, and training: termed “assistive technology services” [27]. Applied to the case of environmental controls, assistive technology services might comprise independent advice supported by clinical reasoning that integrates information about the device itself with the relationship between person, environment, and task, thereby mediating the information asymmetries that disadvantage consumers. To illustrate, one occupational therapist, discussing frail aged clients' use of everyday technologies, stated:

They go to a shopfront and this you-beaut (Australian colloquialism meaning ostensibly great or positive) [sic] thing…someone says “come and buy”… and they do not think about it in the context of their physical ability, their functional ability, their carers or the layout of their home, so you cannot even get it in the door [28].

Hence, while increased utilization of mainstream products has significant benefits, the provision of relevant information and assistive technology services remains important.

## 4. Conclusions

People with disability are using mainstream products such as environmental control systems. The availability of inexpensive, reliable, and interoperable assistive products in mainstream markets has had a positive impact for many people with disability. Assistive products are readily available without the necessity of a prescription, and users are likely to find them more socially and culturally appropriate, as they are indistinguishable from products used by their peers, friends, and family.

The future of assistive technology provision is likely to involve new roles and responsibilities for consumers and occupational therapists. Universal access to assistive technology products and services can only be realized through better connections to other stakeholders and service providers in the health and human services arenas, such as General Practitioners, in-home service providers, pharmacists, and community nurses. Despite working at the interface of consumer need and access to specialised assistive technology provision systems, many of these stakeholders know little about mainstream or specialised assistive products, nor the means of referring to assistive technology services. As one stakeholder described it:

It's a mire out there. It is ridiculous keeping abreast of government changes…huge market out there…it's debilitating not assistive [28].

There are interesting opportunities for occupational therapists to work with people with disability to translate theory into practical problem solving that enhances people's occupational rights and opportunities. The authors propose that Australian assistive technology policies adopt contemporary definitions of assistive products and assistive technology services [6, 29], encouraging occupational therapists to incorporate support for users of mainstream products into their scope of practice. This may not necessitate detailed product knowledge, but clinical reasoning that supports consumer decision-making prior to purchase and application of theory in practical problem solving to customise assistive solutions in situ. Expert users will be increasingly involved, providing the technical knowledge and/or peer support. Continuing professional development will include regular exchanges with people with disability and other stakeholders via networks with special assistive technology interests and skills, facilitated by virtual and local communication and information-sharing platforms that represent an evolution of the Independent Living Centre model.

Individual eligibility for funded supports needs to be based on notions of impairment and disability linked to human rights frameworks (such as the CRPD) [30, 31] rather than a medical model of disability. Equitable access to assistive technology needs to be understood and evaluated by using a capabilities approach [32] that supports the creation of opportunities for individuals to choose from a wide array of assistive technology products and services that addresses needs and outcomes valued by them (in contrast perhaps to those valued by others). This highly individualistic and subjective approach can be made economically efficient only through the use of mainstream as well as specialist assistive products and the decoupling of products and assistive technology services. Rather than rules and lists of eligible assistive products to be funded, individuals need to have the right to purchase widely according to need and personal values. While this enhances consumer rights, it can also be made conditional on increased consumer responsibility and knowledge, given that its operation falls outside previous regulatory frameworks that have “contained” assistive products and has more resonance with individualized funding approaches.

Though mainstream products now meet the needs of many people with disability who would previously have required specialist products and services, they are not universally appropriate, and purpose-built or custom-made products will still be needed and purchased from specialist suppliers and technicians. Additionally, any one product is generally used in combination with other devices and systems, people, and environments, as part of an “assistive solution” [27], and individuals are likely to collect a suite of both mainstream and specialist products and other supports to meet their needs. As above, this underlines the need to define assistive technology broadly as inclusive of specialist and mainstream products and services. The role of occupational therapists must be to support consumers to consider both mainstream and specialised products, collect and use research evidence and economic analyses to demonstrate the effectiveness of this approach, and advocate for policies and funding that facilitate these occupational rights.
